# Eosinophils but not mast cells exert anti-tumorigenic activity, without being predictive markers of the long-term response to Bacillus Calmette-Guérin (BCG) therapy in patients with bladder carcinoma

**DOI:** 10.1007/s00011-025-02028-1

**Published:** 2025-04-24

**Authors:** Ilan Zaffran, Yara Zoabi, Pratibha Gaur, Fidan Rahimli Alekberli, Ekaterini Tiligada, Vladimir Yutkin, Francesca Levi-Schaffer

**Affiliations:** 1 Pharmacology and Experimental Therapeutics Unit, Institute for Drug Research, School of Pharmacy, Faculty of Medicine, The Hebrew University of Jerusalem, Jerusalem, Israel; 2 Department of Pharmacology, Medical School, National and Kapodistrian University of Athens, 11527 Athens, Greece; 3 Department of Urology, Hadassah Hebrew University Medical Center, Jerusalem, Israel

**Keywords:** Mast cells, Eosinophils, Tumor micro-environment, Bladder cancer, BCG

## Abstract

**Background:**

Bacillus Calmette-Guerin (BCG) therapy is an established immunotherapy for non-muscle invasive bladder cancer (NMIBC); however, the response variability of patients remains a challenge, necessitating insight into immune cell function. Previous studies established that a preexisting Th2 immune microenvironment correlates with a positive BCG therapy outcome. Therefore, in this study, we explored the role of mast cells (MCs) and eosinophils in bladder cancer as a potential predicting tool for BCG immunotherapy response.

**Methods:**

We investigated the effect of MCs and eosinophils on bladder cancer cell viability together with their chemotactic migration towards cancer cells in vitro. The effect of BCG on these immune cells was also evaluated. Moreover, we performed an orthotopic model of bladder cancer in MC- and eosinophil-deficient mice. Finally, to evaluate whether these immune cells predict the therapy response, 26 patient biopsies pre-BCG treatment were analyzed for MC and eosinophil numbers in the tissue and sequenced for gene expression.

**Results:**

Eosinophils, but not MCs, reduced bladder cancer cell viability in vitro and inhibited tumor growth in vivo. However, addition of BCG did not increase these effects in vitro. Patient biopsy analysis and mRNA sequencing showed that neither cell type predicted long-term therapy responsiveness. Gene expression analysis suggested that extracellular matrix and epithelial-to-mesenchymal transition factors could influence BCG therapy outcomes.

**Conclusion:**

Even though eosinophils exhibit anti-tumorigenic effects in bladder cancer, neither MCs nor eosinophils were predictive of the long-term BCG therapy response. However, our findings implicate that matrix-related factors may modulate BCG therapy responses.

**Supplementary Information:**

The online version contains supplementary material available at 10.1007/s00011-025-02028-1.

## Introduction

Mast cells (MCs) and eosinophils are innate immune cells primarily associated with allergic diseases and T-helper cell 2 (Th2) immunity. Nevertheless, in the last decades, intense investigation on their roles in the tumor microenvironment (TME) has shown that they modulate various events of tumor biology, such as cell proliferation and survival, angiogenesis, and metastasis [1, 2].

In bladder cancer, MCs are mostly associated with pro-tumorigenic functions and poor prognosis [3]. On the other hand, eosinophils have been demonstrated in vitro to induce cytotoxicity against a broad range of human cancer cell lines and to retard xenograft tumor growth of certain types of human cancers [4, 5].

Bladder cancer is characterized by relatively high incidence of recurrence [6]. In non-muscle-invasive urothelial bladder cancer (NMIUBC), intravesical therapies with Bacillus Calmette–Guérin (BCG) is the main treatment to prevent progression of the tumor after initial trans-urethral resection of bladder tumor (TURBT). However, the responsiveness to BCG treatment remains low (~ 40%) [7]. Therefore, additional and/or alternative therapies are needed for the management of the disease in non-responsive to BCG patients. Even though the mechanism of action of BCG is not fully elucidated [8], it is thought to act by inducing an acute and local T-helper cell 1 (Th1) immune response in the bladder wall, involving numerous inflammatory cytokines and immune cells such as T and natural killer (NK) cells [9, 10]. Importantly, the polarization towards Th1 or Th2 immunity has been described to influence the response to BCG, a pre-existing Th2 response being a benefit for the response to BCG [8, 11, 12].

Consequently, the role of the immune system in non-muscle invasive urothelial carcinoma (NMIUC) and particularly that of Th2-associated cells, is of particular interest. In this study, we investigated the role of human cord blood derived MCs (CBMCs) and peripheral blood eosinophils (pbEos) in bladder cancer, both in vitro and in an in vivo orthotopic murine model of bladder cancer. Furthermore, the number of MCs and eosinophils was evaluated in twenty-six biopsies collected from patients diagnosed with primary NMIUC prior to an intravesical injection of BCG, to explore whether the presence of these immune cells in the tissue may influence and/or be predictive of the tumor response to BCG.

## Results

### Eosinophils but not MCs are anti-tumorigenic in vitro, but they both migrate towards bladder cancer cells

**Fig. 1:**
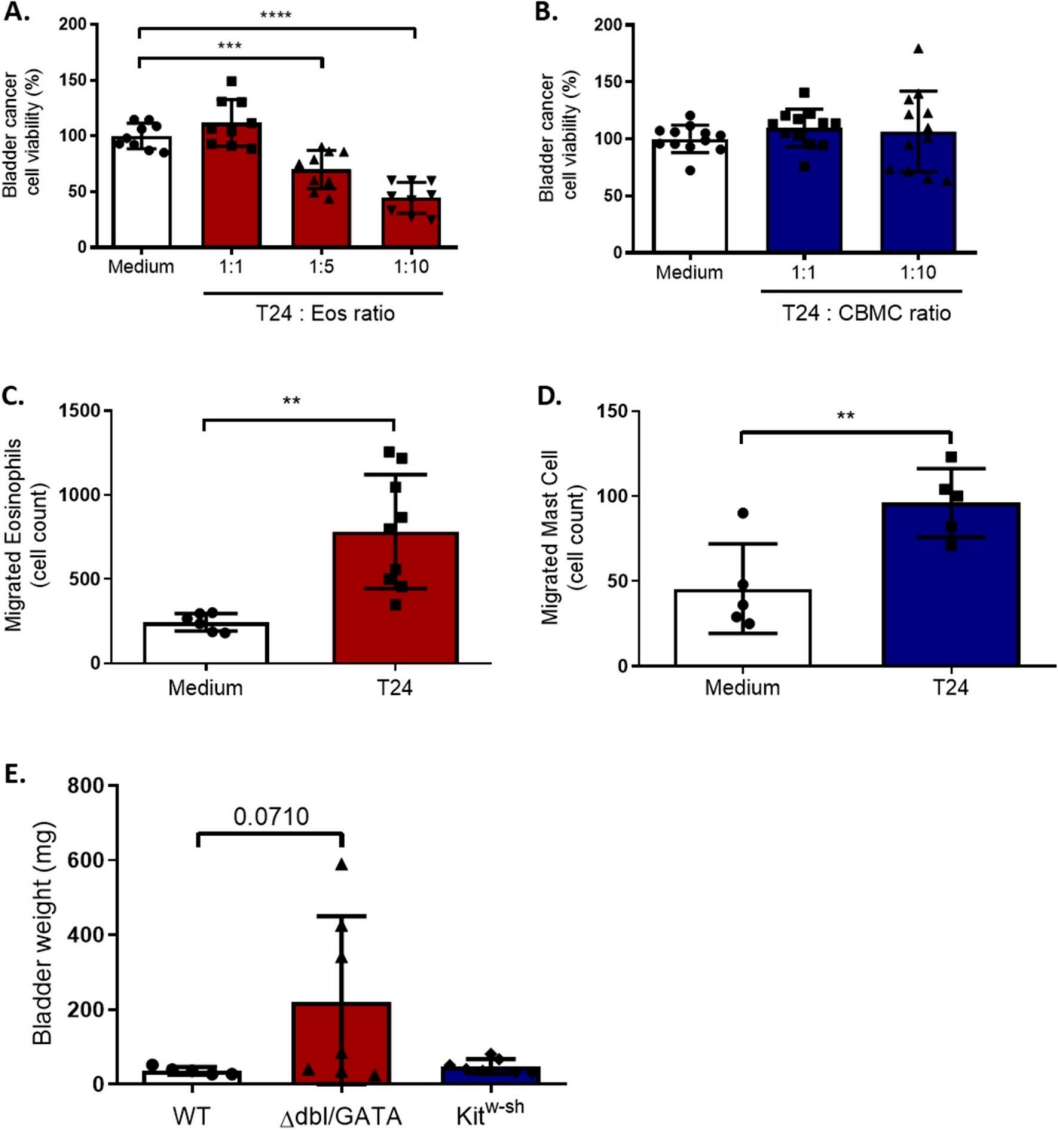
Eosinophils but not MCs are anti-tumorigenic in vitro, but they both migrate towards bladder cancer. **A** and **B** T24 cell viability assessed by MTT. T24 cells co-cultured with peripheral blood eosinophils or cord blood derived mast cells (CBMCs) at indicated ratios for 72 h; n = 3. **C** and **D** Eosinophil and CBMC migration in response to T24 cell line. FITC-labelled CBMCs and eosinophils were cultured in transwell plate with T24 cells for 20 h. Migrated cell number was determined by flow cytometry. n = 3 and n = 4 respectively for MCs and eosinophils. **E** Tumor weight (g) in the bladder of wild type (WT; n = 5), GATA (Δdbl/GATA; n = 7), and Sash (Kit^w−sh^; n = 7) mice; in a single experiment

To assess the role of MCs and eosinophils in bladder cancer, we incubated the transitional cell carcinoma (TCC) cell line T24 with pbEos or CBMCs at different ratios for 72 h and determined the bladder cancer cell viability by the MTT assay. Similarly to previous reports [13] our data showed that pbEos decreased the T24 cell viability up to 55% at the highest eosinophil/T24 ratio (Fig. 1A), whereas CBMCs did not affect T24 viability (Fig. 1B). Notably, the decrease of the bladder cancer cell viability was lower when eosinophils were incubated with the low grade TCC (RT112) rather than with the high grade (T24) (15.2 ± 4.8% and 55 ± 5.9%, respectively; Supplementary Fig. S1).

Moreover, using a transwell assay we found that after 20 h of incubation, the numbers of migrated CBMCs and pbEos significantly increased in the presence of T24 cells (Fig. 1C, D).

The role of MCs and eosinophils was then evaluated in an in vivo orthotopic model of bladder cancer, using wild type (WT), MC (Kit^w−sh^, Sash) and eosinophil (ΔdblGATA; GATA) deficient mice. In line with our in vitro data, eosinophils but not MCs displayed a non-significant trend of anti-tumorigenic activity evidenced by the increased tumor weight of the bladder of GATA mice in comparison to the wild type (WT) mice (220.2 ± 86.9 vs. 35.9 ± 4.5 mg), while tumor weight was comparable in WT and Sash mice (35.9 ± 4.5 and 47.9 ± 7.4 mg; Fig. 1E).

### BCG induces eosinophil and MC activation but does not influence their migration and the anti-tumor activity

**Fig. 2:**
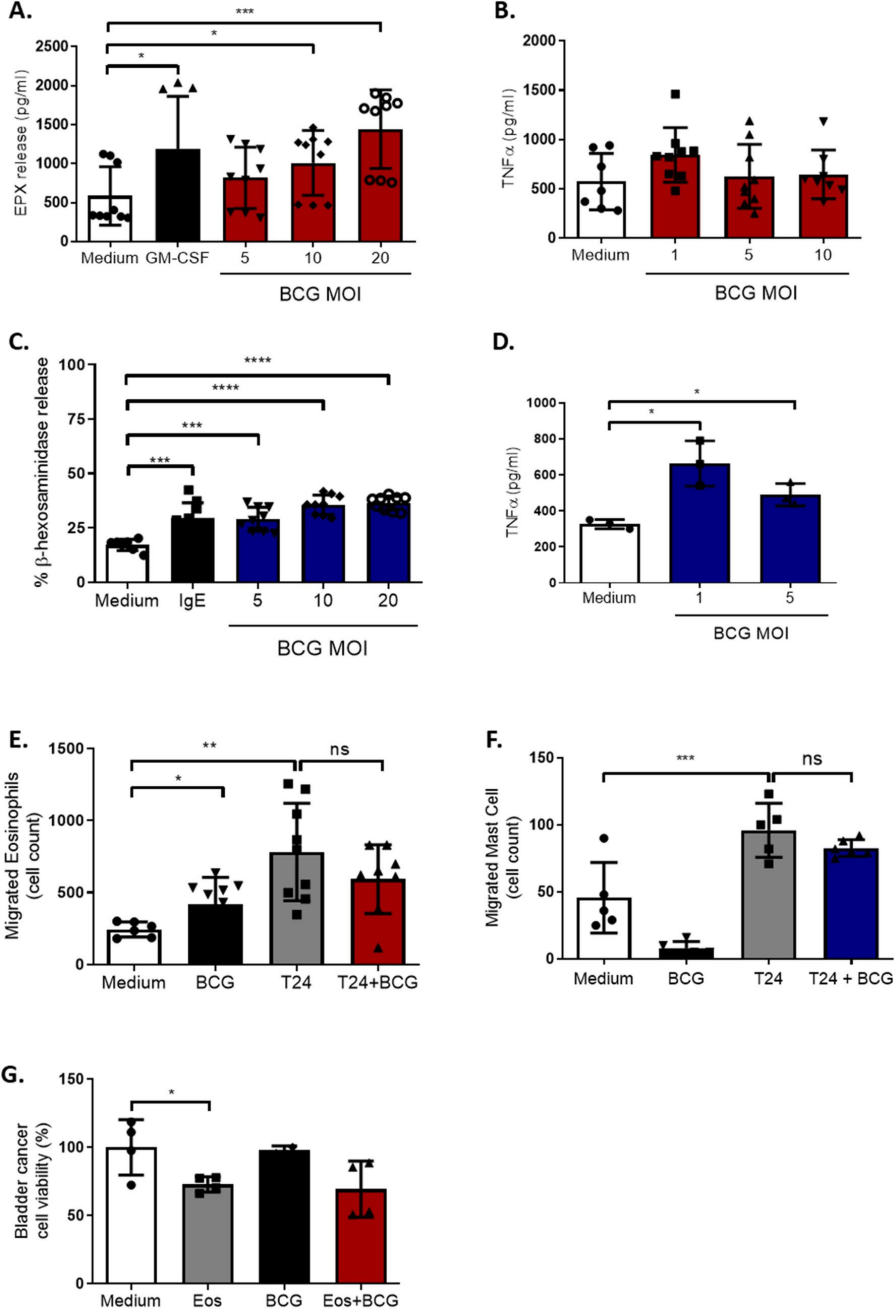
BCG induces eosinophils and MCs activation but does not influence the migration and the anti-tumor activity of these cells. **A** and **B** Eosinophil degranulation and tumor necrosis factor (TNF)-α release in response to BCG. Eosinophil peroxidase (EPX) and TNF-α levels were measured from eosinophils co-cultured with BCG at indicated ratios for 1 and 4 h respectively; n = 4 for EPX and n = 3 for TNF-α level. **C** and **D** CBMC degranulation and TNF-α release in response to BCG. β-hexosaminidase and TNF-α levels were measured upon release from CBMCs co-cultured with BCG at indicated ratios for 1h and 4h, respectively; n = 3. **E** and **F** Eosinophil (**E**) and CBMC (**F**) migration in response to T24 cells and BCG. FITC-labelled CBMC and eosinophil were cultured for 20 h in a transwell plate in presence of T24 cells, BCG (MOI 10), or both together. Migrated cell number was determined by flow cytometry; n = 3. **G** T24 cell viability assessed by MTT. T24 co-cultured with eosinophils (Eos), BCG (MOI 10), or both together for 72 h; n = 2

To further explore the effect of BCG on MCs and eosinophils, we co-cultured these cells with BCG and evaluated cell degranulation by measuring the release of eosinophil peroxidase (EPX) and β-hexosaminidase as a marker of activation of eosinophils and MCs respectively. Moreover, the release of tumor necrosis factor (TNF)-α was assessed in the respective supernatants. PbEos and CBMCs cultured in the presence of BCG were found to degranulate, as shown by the increases in EPX and β-hexosaminidase levels (Fig. 2A–C). However, MCs but not eosinophils released TNF-α when incubated with BCG (Fig. 2B–D).

Next, we tested the capacity of BCG to increase the migration rate of CBMCs and pbEos toward T24 cells. BCG alone was found to induce the migration of pbEos (Fig. 2E), but not of CBMCs (Fig. 2F). Interestingly, the chemotactic effect of BCG was not additive to that of T24, since the migration of pbEos or of CBMCs incubated with BCG primed-T24 was similar to the control cultures performed in the absence of BCG. Additionally, the addition of BCG to the pbEos/T24 cultures did not increase the tumoricidal effect of the eosinophils, as shown by the MTT assay (Fig. 2G).

### Eosinophils and MCs in the bladder microenvironment do not predict the response to BCG therapy

To evaluate whether MC and eosinophil presence in the bladder microenvironment, prior to BCG treatment, could be predictive of the response to the treatment, bladder biopsies from 26 patients diagnosed with primary NMIUBC (Table 1) were collected after TURBT and prior to intravesical injection of BCG. Specimens were separated in two groups according to the progress of the disease two years after the patients had completed a standard 6-weekly intravesical instillation with BCG. Patients were classified as responders if there was no recurrence of the disease during the follow-up based on cystoscopy, cytology, and urine test 2 years after the BCG therapy. In contrast, non-responders had a positive biopsy for TCC 2 years following BCG injections.

**Table 1:** Demographic characteristics of the bladder cancer patients

Patient ID	Age	Gender	Diagnosis	GRADE	Treatment	Outcome 2 years post treatment	Response status
1	52	F	Papillary TCC, non-invasive	3	BCG	Not recurrent	Y
2	84	M	Papillary TCC. No signs of invasion to lamina prupria. Muscle with no signs of invasion	High grade	BCG	Not recurrent	Y
3	69	M	Papillary urothelial carcinoma, high grade, with focal invasion into the lamina propria and involvement of von Brunn glands. Muscle tissue is free. Background foci suspicious for carcinoma in situ seen	High grade	BCG	Recurrent	N
4	80	M	Non-invasive high grade papillary urothelial carcinoma Multifocal urothelial carcinoma in situ. Muscularis propria is not identified	High grade	BCG	Not recurrent	Y
5	75	M	Papillary urothelial carcinoma, high grade, non-invasive Muscularis propria is present, uninvolved/Papillary urothelial carcinoma, low grade, non-invasive, with extensive necrosis. Focal urothelium with intestinal metaplasia	High grade	BCG	Recurrent	N
6	66	M	Non-invasive high grade papillary urothelial carcinoma. Urothelial carcinoma in situ	High grade	BCG + Synergo	Not recurrent	Y
7	77	M	Non-invasive high-grade papillary urothelial carcinoma	High grade	BCG	Recurrent	N
8	61	M	Papillary transitional cell carcinoma, high grade, non-invasive	High grade	BCG	Recurrent	N
9	71	M	Fragments of poorly preserved papillary urothelial carcinoma with no evidence of invasion. Papillary urothelial carcinoma high grade, with focal inverted growth and focal superficial invasion into the lamina propria. Invasive high-grade papillary urothelial carcinoma. Tumor invades the lamina propria	High grade	BCG	Recurrent	N
10	72	M	Urothelial carcinoma, high grade, with lamina propria invasion. Background focal urothelial carcinoma in situ is seen	High grade	BCG	Not recurrent	Y
12	71	M	Urinary bladder mucosa with carcinoma in situ and early papillary formation. Invasive high-grade papillary urothelial carcinoma, Tumor invades the lamina propria	High grade	BCG	Recurrent	N
13	81	F	Papillary transitional cell carcinoma, high grade (WHO grade 3) invading into the lamina propria and seen in muscularis mucosa. Vascular invasion found. There is no muscularis propria in the specimen	3	BCG	Not recurrent	Y
15	62	M	Fragments of papillary transitional cell carcinoma, high grade (WHO grade III)	3	BCG	Not recurrent	Y
16	85	M	Superficial fragments of transitional cell carcinoma, papillary high grade (WHO grade 3)	3	BCG + MMC + Radiation	Recurrent	N
17	82	F	Papillary transitional cell carcinoma, high grade (WHO grade 3) focal invasion into the lamina propria is found	3	BCG	Recurrent	N
18	46	M	Papillary transitional cell carcinoma, high grade (WHO grade 3), non-invasive	3	BCG + Synergo	Recurrent	N
19	86	M	Papillary transitional cell carcinoma of urinary bladder, high grade (WHO grade 3) with extensive inverted growth, non-invasive	3	BCG + Synergo + Radiation	Recurrent	N
20	81	M	Fragments of papillary transitional cell carcinoma, high grade (WHO grade 3) with focal inverted growh with focal superficial invasion into the lamine propria	3	BCG	Not recurrent	Y
21	69	M	Papillary urothelial carcinoma of bladder high grade, with invasion into lamina propria and suspicious vascular invasion. There is focal inverted growth. There is focal tumor necrosis. The detrusor muscle is free of tumor	High grade	BCG	Not recurrent	Y
22	82	M	Papillary transitional cell carcinoma, high grade, suspicious for superficial invasion into the subepithelium and the lamina propria. There is a small fragment of urothelium with carcinoma in situ	High grade	BCG	Not recurrent	Y
23	75	M	Fragments of high grade papillary transitional cell carcinoma, with inverted growth pattern, invading lamina propria. Vascular invasion is seen	High grade	BCG	Recurrent	N
24	87	M	Carcinoma in situ and high grade urothelial carcinoma, papillary and solid, infiltrating lamina propria, Papillary transitional cell carcinoma, high grade, with focal inverted growth pattern and invasion into the lamina propria. Detrussor muscle in not involved	High grade	BCG	Recurrent	N
25	66	M	Superficial fragments of papillary transitional cell carcinoma, high grade, with foci of micropapillary differentiation and focal inverted growth pattern	High grade	BCG	Not recurrent	Y
26	74	M	Papillary urothelial carcinoma, infiltrated to lamina propria	High grade	BCG	Not recurrent	Y
28	79	M	Papillary urothelial carcinoma, high grade, with inverted growth pattern. Focal supeficial invasion into the lamina propria	High grade	BCG	Recurrent	N
29	87	M	Superficial fragments of non-invasive high-grade papillary urothelial carcinoma. Urothelial carcinoma in situ, involving Von Brunn's nests. High-grade papillary urothelial carcinoma invading the lamina propria, Angiolymphatic invasion seen. High-grade urothelial carcinoma invading the lamina propria/prostatic stroma	High grade	BCG + Radiation	Not recurrent	Y

TCC—Transitional cell carcinoma; BCG—Bacillus Calmette Guerin; MMC—mitomycin C

**Fig. 3:**
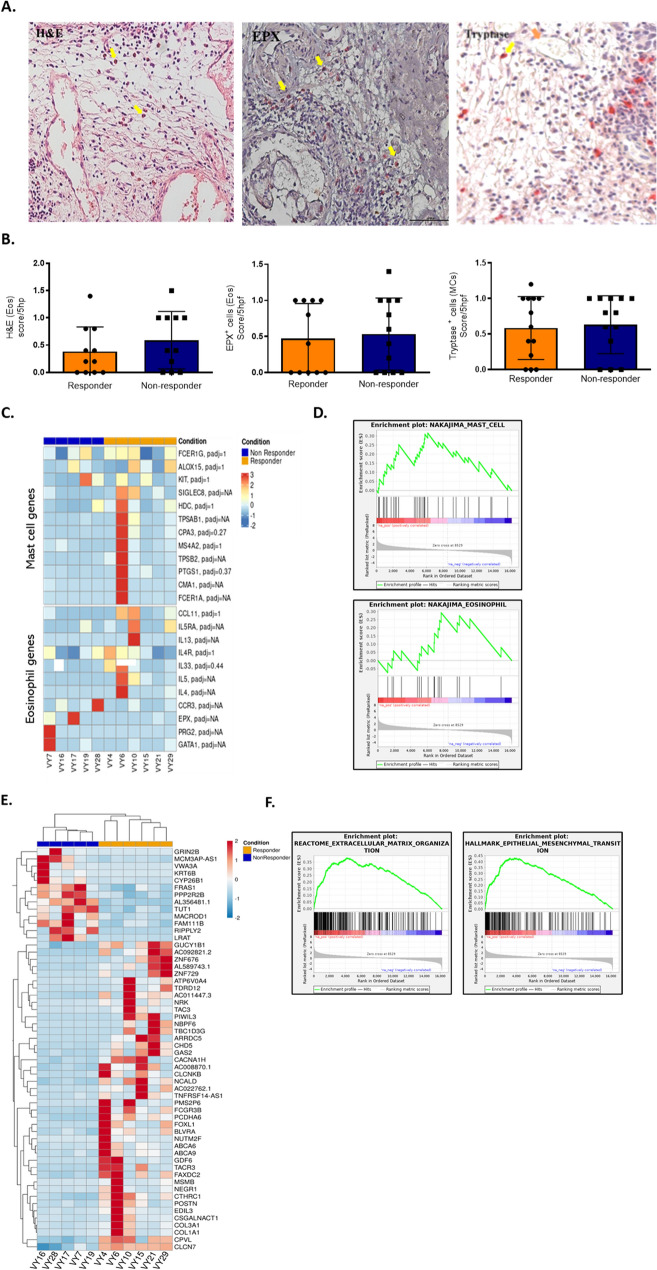
Eosinophils and MCs in the bladder microenvironment do not predict the response to BCG therapy. **A** Eosinophils and MCs staining in human tissues. Eosinophils were assessed by hematoxylin and eosin (H&E; left panel), or anti–EPX (middle panel) staining. MCs were assessed using anti-tryptase (right panel). Yellow arrows indicate eosinophils or MCs, and orange arrows indicate tissue blood vessels. Images were acquired using Nikon TL microscope at X200 magnification. **B** Eosinophil and MC score in the tissue from responder and non-responder patients as evaluated in A. Patients who received BCG immunotherapy were divided in two groups based on recurrence (not responder; n = 12 and n = 13 for eosinophils and MCs, respectively) of the disease or remaining disease free (responder; n = 11 and n = 13 for eosinophils and MCs, respectively) following two years follow up. Mann–Whitney test was used to analyze the data. **C** Heat map of MCs and eosinophils related genes in responder and non-responder patients. Normalized expression values were scaled at gene level (scale is shown at top-right). Both genes (rows) and samples (columns) were hierarchically clustered. **D** Gene Set Enrichment Analysis (GSEA) of MCs and eosinophils related genes. MCs and eosinophils do not display a change between responders and non-responders. **E** Heat map of the 56 differentially expressed genes in responder and non-responder patients. Normalized expression values were scaled at gene level (scale is shown at top-right). Both genes (rows) and samples (columns) were hierarchically clustered. **F** GSEA of epithelial-mesenchymal transition (EMT), extracellular matrix (ECM) pathways from the same cohort. EMT and ECM organization pathways were upregulated in responders compared to non-responders

Eosinophils and MCs stained with hematoxylin and eosin (H&E), anti-EPX antibody (eosinophil staining, Fig. 3A left and middle panel), and anti-tryptase antibody (MCs staining, Fig. 3A right panel) were counted in the specimens. No difference in eosinophil or MC numbers (Fig. 3B) were observed between responding and non-responding patients.

In line with the histological cell count, the gene expression analysis of mRNA from 11 samples (5 non-responders and 6 responders) showed that the expression of genes related to eosinophils and MCs were comparable in the samples of the two groups (Fig. 3C). In addition, the performed gene set enrichment analysis (GSEA) revealed that the gene sets related to MCs and eosinophils were comparably expressed in the biopsies obtained from both responders and non-responders (Fig. 3D). Furthermore, no differences were observed in genes that are related to innate immune system between responders and non-responders (Supplementary Fig. S2A).

Nevertheless, we revealed 56 significantly dysregulated genes between the responding and non-responding groups of patients (Fig. 3E). Among these genes, we detected several related to matrix organization and epithelial to mesenchymal transition (EMT). For example, *FRAS1* was found to be downregulated while *COL1A1* and *COL3A1* genes were upregulated in bladder biopsies of BCG responder patients. Moreover, a positive correlation was found in the GSEA analysis of extracellular matrix organization and EMT pathways in responder patients (Fig. 3F and Supplementary Fig. S2B).

Altogether, these data implicate a functional contribution of the initial bladder structure rather than of the immune microenvironment in the response to BCG.

## Discussion

MCs and eosinophils are innate immune cells associated with allergic reactions and Th2 immunity. However, their roles extend to tumor biology where they influence cell proliferation, survival, and metastasis. While some studies suggest that a pre-existing Th2-polarized TME may correlate with improved BCG response due to the Th2-to-Th1 shift induced by BCG, the role of eosinophils and MCs in bladder cancer remains debated [11, 12]. In this study we aimed to assess their contribution to tumor progression in response to BCG response.

Eosinophils have been shown to infiltrate various tumors, including melanoma and colon cancer, with both pro- and anti-tumorigenic roles [2]. They may exert direct cytotoxic effects on cancer cells by releasing granules containing EPX, eosinophil cationic protein (ECP), and major basic protein (MBP), as well as inflammatory cytokines (TNFα, IL-18) [4, 13] and granzyme A [14], which induce tumor cell apoptosis. In our study, eosinophils migrated toward bladder cancer cells and significantly reduced tumor cell viability, particularly in high-grade TCC. This suggests a potential tumor-suppressive role where eosinophils’ presence could enhance anti-tumor immunity.

However, eosinophils may also promote tumor progression through their ability to release cytokines and growth factors such as IL-4, IL-5, and vascular endothelial growth factor (VEGF), which can support angiogenesis and tumor evasion [15]. Moreover, eosinophils’ presence in the TME activate indoleamine 2,3-dioxygenase 1 (IDO1) and produce L-kynurenine to suppress cytotoxic T cell function and thus support immune suppression [16]. Some studies reported that high eosinophil counts in blood or urine are associated with poor BCG response [17, 18]. Additionally, quantitative assessment of tumor-associated tissue eosinophilia (TATE) in tumor stroma has shown significantly higher TATE in primary urothelial carcinomas tending to recur [19], possibly reflecting an immunosuppressive phenotype that promotes tumor persistence. In contrast, other studies showed that eosinophil infiltration at diagnosis predicts a favorable BCG response [8, 11, 12], suggesting context-dependent roles that warrant further investigation.

MCs exhibit similarly complex functions in cancer. They accumulate in bladder carcinomas and are implicated in tumor angiogenesis, tissue remodeling, and immune modulation [20]. While they migrated toward bladder cancer cells in our study, they did not significantly alter tumor cell viability. Their impact appears to be more indirect, likely through the secretion of pro-angiogenic and immunomodulatory factors. MC-derived histamine, tryptase, and chymase can promote tumor growth by stimulating angiogenesis and extracellular matrix remodeling. Conversely, MCs can also exhibit anti-tumor properties by releasing TNF-α, granzyme B [21], and IL-17, which enhance anti-tumor immunity. A clinical study reported increased IL-17 + MCs in carcinoma in situ (CIS) patients following BCG therapy, potentially contributing to immune activation [22].

In the present study BCG was found to induce degranulation in both MCs and eosinophils, with MCs specifically releasing TNF-α. These findings align with previous reports showing murine or human MC degranulation upon exposure to *M. tuberculosis* and BCG [23, 24] and eosinophil degranulation triggered by live BCG [25]. Despite this activation, BCG exposure did not enhance MCs and eosinophils chemotaxis toward bladder cancer cells or increased eosinophil-mediated cytotoxicity. This suggests that while BCG triggers immune activation, its direct effect on these cells may not be a key determinant of therapeutic response.

Although eosinophils and MCs respond to BCG, their presence in the bladder of patients before treatment did not predict BCG efficacy in our patient cohort. This contrasts with studies linking eosinophil infiltration to positive BCG outcomes [8]. It is noteworthy that these studies were performed on patients only up to 6 weeks after completion of the BCG induction course. This is a relative short time to get a diagnostic response and it may possibly explain the difference with our observations. Nevertheless, our findings align with findings that high eosinophil counts in blood may indicate therapy failure [17, 18].

Beyond immune cell infiltration, our transcriptomic analysis revealed dysregulated genes (FRAS1, COL1A1, COL3A1) related to extra cellular matrix (ECM) remodeling and EMT. ECM components can influence immune cell infiltration and activation, suggesting that TME structural dynamics may play a more decisive role in BCG response than eosinophil or MC presence alone.

Our study underscores the dual roles of eosinophils and MCs in bladder cancer, where they can exert both pro- and anti-tumor effects depending on the context. While eosinophils demonstrated cytotoxic activity against bladder cancer cells, their overall impact on BCG response is not yet fully understood. MCs, despite their known immunomodulatory functions, appeared to have a limited direct effect on tumor cell viability. Importantly, ECM remodeling and EMT-related processes may be more crucial in determining BCG response, highlighting the need for further research to clarify the interplay between immune cells and the TME.

## Materials and methods

### Cells and cell cultures

Human bladder cancer cell lines (T24 and RT112) and the murine bladder carcinoma cell line MB49-luc (a kind gift from Prof. Gilad Bachrach, The Hebrew University-Hadassah School of Dental Medicine, Jerusalem, Israel) were cultured at 37 °C in 5% CO_2_ in Dulbecco's Modified Eagle Medium (DMEM; Gibco-ThermoFisher, Waltham, MA, USA) supplemented with 10% (v/v) fetal calf serum (FCS; HyClones; Utah, USA), penicillin (100IU/ml), streptomycin (100 µg/ml; Biological Industries, Beit Haemek, Israel), and 1% (v/v) sodium pyruvate (Biological Industries, Beit Haemek, Israel), denoted throughout the paper as “culture medium”.

### Bacterial cultures

The *M. bovis* BCG was prepared after reconstitution in phosphate buffer saline (PBS) of freeze-dried preparation containing BCG (2–8 × 10^8^ CFU/Vial; OncoTICE; Meck Sharp and Dohme, Petah-Tikva, Israel). The concentration was then adjusted as indicated in the figure legends.

### Human peripheral blood eosinophil purification

Human eosinophils were purified as previously described [26] from the peripheral blood of asymptomatic mildly atopic volunteers (blood eosinophil levels 5–10%) not taking any medication. Written informed consent was obtained according to the guidelines of the Hadassah-Hebrew University Human Experimentation Helsinki Committee (0410-14-HMO). Venous blood (150 ml) was collected in heparinized syringes and left to sediment in 6% dextran (Sigma-Aldrich, Jerusalem, Israel). Leukocytes were loaded on lymphoprep solution (STEMCELL Technologies; Vancouver, Canada) and centrifuged (25 min; room temperature (RT); 1400 rpm with no brakes). Neutrophils and contaminating lymphocytes were tagged in the granulocyte-enriched pellet with micromagnetic beads bound to anti-CD16 and anti-CD3 (Miltenyi Biotec, North Rhine-Westphalia, Germany). Eosinophils were purified by passing the cell suspension through a magnetic column (LS-MACS). Eosinophils were evaluated by Kimura staining and by flow cytometry using APC-anti-human CCR3 (Abcam, Cambridge, UK) and PE anti-human Siglec-8 (BioLegend, California, USA) and collected at a purity of > 90%, with viability of > 98% (trypan blue staining). Then, eosinophils were re-suspended in eosinophil medium consisting of RPMI-1640 supplemented with 10% heat-inactivated FCS, penicillin–streptomycin solution (100 u/ml; Biological Industries), and granulocyte–macrophage colony-stimulating factor (GM-CSF; 5 ng/ml; Peprotech, Rocky Hill, NJ, USA).

### Human cord blood derived mast cells purification

CBMCs were obtained as previously reported [27]. Human umbilical cord blood was obtained from normal births after informed consent and according to the guidelines of the Hadassah-Hebrew University Human Experimentation Helsinki Committee (7-14.01.05). CD34^+^ progenitor cells were enriched by lymphoprep centrifugation and grown in MEM-α medium supplemented with 2 mM L-glutamine, ribonucleases (10 µg/mL; Biological Industries), stem cell factor (SCF; 100 ng/ml; Peprotech), IL-6 (10 ng/mL; Peprotech), and PGE2 (0.3 µM; Sigma-Aldrich).

CBMCs were harvested for the experiments between 7 and 9 weeks of culture when > 95% of the cells were stained metachromatically with toluidine blue staining.

### Bladder cancer viability assay

As a marker of cell viability, bladder cells metabolic rate/mitochondrial activity was determined by 3-(4,5-dimethylthiazol-2-yl)-2,5-diphenyltetrazolium bromide (MTT; Sigma-Aldrich). T24 or RT-112 cells (5 × 10^3^ cells/100 µl/well) were incubated overnight in a 96-well flat bottom plate (ThermoFisher, Nunc, Kiryat Shmona, Israel). The medium was then replaced with fresh culture medium containing CBMCs or eosinophils in 1:1, 1:5 and 1:10 ratio. The plate was then incubated at 37 °C, 5% CO_2_ for additional 48 h. Following the incubation period, CBMCs and eosinophils in suspension were removed, and the adherent cancer cells were washed once with PBS and incubated for additional 2.5 h in a fresh culture medium containing 10 µl MTT. Then, the plate was centrifuged (1250 rpm, 5 min, 4 °C), the medium was discarded and 100 µl dimethyl sulfoxide (DMSO; Sigma-Aldrich) was added to dissolve the crystals. Optical density (O.D.) was measured at 565 nm in an ELISA reader (BIO-TEK, 185 Winooski, VT, USA).

### Chemotaxis assay

The chemotaxis assay was performed using a 5 µm transwell plate (Getter Biomed, Israel). The lower chamber was seeded with T24 cells (2 × 10^5^/well/750 µl) in their medium and incubated overnight at 37 °C, 5% CO_2_ and the upper chamber was loaded with fluorescein isothiocyanate (FITC)-labelled CBMCs or pbEos (3 × 10^5^/well/300 µl). Briefly, MCs and eosinophils were stained with 1 µl FITC/10^5^ cells for 30 min at RT, washed twice with PBS and resuspended in 300 µl/2 × 10^5^ cells. Subsequently, the plate was incubated in 37 °C, 5% CO_2_ for 24 h to allow migration across the membrane in response to full medium only or to cells with medium. Twenty-four hours later, the filter inserts were removed and 250 µl was aspirated from the lower chamber. Then the number of MCs and eosinophils migrated into the lower chamber was quantified by flow cytometry, by counting the number of FITC^+^ cells during 30 s. For chemotaxis assay with BCG and bladder cancer cells, the BCG (MOI 10) was added simultaneously with the CBMCs and eosinophils in the upper chamber.

### Orthotopic bladder cancer model

The orthotopic bladder cancer model was performed according to the previously described protocol [28]. Nine to 14-week-old female wild type C57/BL6, MC (Kit^w−sh^; Sash)—and eosinophil—(ΔdblGATA; GATA) deficient mice (both from C57/BL6 background) were maintained at our animal care facility for 1 week prior to use. Mice were anesthetized by i.p injection of 85 mg/kg ketamine/15 mg/kg xylazine diluted in 1:1 with PBS.

To ensure an efficient laser coagulation, an area of 1 cm^2^ was shaved on the backs of the mice and a 24-gauge Teflon i.v. catheter (Becton Dickinson, New Jersey, United States) was inserted transurethrally into the bladder using paraffin. Mice were then placed with their backs on the ground plate of the cautery unit. To optimize contact, we used electrocardiogram electrode contact gel (Covidien; Dublin, Ireland). The soft tipped end of a spring-wire guide of a 24-gauge central venous catheter (Shuyou, Willich, Germany) was inserted into the bladder via the Teflon catheter and gently pushed forward until it reached the bladder wall. The guide wire was attached to the cautery unit and a monopolar coagulation was applied for 5 s at the lowest setting (5 W). After removal of the guide wire, 100 µl of PBS containing 3 × 10^4^ murine bladder carcinoma cell line transfected with luciferase (MB49-luc) was instilled. The catheters were pinched off with a clamp, kept locked and left in place until the mice awakened (Supplementary Fig S1B left panel). Using this method, we ensured a dwell time of 2–3 h. Ten days after tumor inoculation, mice were injected i.p with D-luciferin (Sigma) and analyzed by bioluminescence imaging (IVIS). Mice in which no tumor was observed were removed from the experiment (Supplementary Fig. S1B right panel). After 40 days mice were sacrificed, and the bladder tumor was extracted and weighted. All experiments were performed according to the guidelines of the Hebrew university ethics committee for animal studies (MD-20-15950-5).

### Eosinophil degranulation assay

PbEos (1 × 10^5^/100 µl) were re-suspended in the specific medium/buffer according to the degranulation assay performed (see below). The cells were incubated with BCG (MOI of 1, 5, 10) in 96-well plates (37 °C, 45 min, 5% CO_2_). As positive control, eosinophils were incubated with granulocyte–macrophage colony-stimulating factor (GM-CSF; 100 ng/ml) at 37 °C for 20 min.

For EPX release, eosinophils were re-suspended in 0.1% BSA/PBS and incubated with BCG. EPX release was measured by a colorimetric assay using a standardized assay with purified human EPX (a kind gift of Prof. Gerald J. Gleich, University of Utah Health Sciences Center, Salt Lake City, UT, USA) and freshly prepared peroxidase substrate solution containing o-phenylenediamine (OPD; ThermoFisher). Data are expressed as EPX concentration, based on a standard curve.

At all-time points, an aliquot of infected cells was harvested and counted. This step allowed an exact quantification of cells as well as the determination of cellular viability by trypan blue exclusion. Recovery of cells was 80% in all experiments, with cell viability regularly exceeding 90% of total cells.

### Mast cell degranulation assay

For activation of CBMCs, human myeloma IgE (0.3 µg/mL, Sigma‐Aldrich, #401152) was used for sensitizing the cells in the presence of recombinant human IL‐4 (Gibco, PeproTech, #200-04; 10 ng/mL) for 3 days. Subsequently, cells were washed twice and resuspended in Tyrode's buffer (consisting of 137 mM NaCl, 5.5 mM glucose, 2 mM KCl, 12 mM NaHCO_3_, and 0.3 mM Na_2_HPO_4_, supplemented with 1.8 mM CaCl_2_ and 0.9 mM MgCl_2_; pH 7.3. CBMCs were then incubated for 1 h with polyclonal rabbit anti-human IgE Ab (5 µg/mL, Dako, #A0094) as positive control or with BCG at MOI of 1, 5, and 10.

To measure the degranulation of CBMC, we measured the release of β-hexosaminidase as a readout. The supernatant was collected by centrifugation and the remaining cell pellet was lysed in Tyrode’s buffer containing 10% NP-40. Ten microliters of pellet and supernatant were incubated with 50 µL of substrate solution (1.3 mg/mL *p*-Nitrophenyl-N-acetyl-β-D-glucose in 0.1 M sodium citrate, pH 4.5).

The measurement of *p*-nitrophenol, generated by β-hexosaminidase was done by a spectrophotometric reader (BioTek Eon) at a wavelength of *λ* = 405 nm. The amount of degranulation in percent was determined as follows:

$$\text{Degranulation }[\%]= \frac{\text{OD suppernatant}}{\text{OD supernatant }\times \text{OD lysate}} \times 100$$

At all-time points, an aliquot of infected cells was harvested and counted. This step allowed an exact quantification of cells as well as the determination of cellular viability by trypan blue exclusion. Recovery of cells was 80% in all experiments, with cell viability regularly exceeding 90% of total cells.

### Evaluation of TNF-α release

PbEos and CBMCs (1 × 10^5^/100 µl) were incubated with BCG (MOI of 1, 5, and 10) for 4 h (37 °C, 5% CO_2_) in 96-well plates. Following centrifugation at 250 g supernatants were collected and TNF-α levels were determined using commercial enzymatic immunoassay kits according to the manufacturer's instructions (Cat. No: 900-T25, Peprotech, Rocky Hill, NJ, USA).

### Primary NMIUC patient samples

Formalin fixed paraffin embedded (FFPE) tissue sections and H&E-stained specimens obtained from each patient diagnosed with primary NMIUC of the bladder were obtained from the department of Urology of Hadassah Ein-Kerem Hospital (Israel). All the patients included in the study had completed 6 weekly intravesical injection with BCG following TURBT resection. The patients were followed up in the Urology Clinic according to the best treatment practice on a routine basis for at least 2 years after the first TURBT surgery. Patients who received immunosuppressive drugs were excluded from the study.

### Immunohistochemistry (IHC) staining of MCs

Twenty six bladder sections were deparaffinized by immersing the slides through the following solutions: (1) xylene; two washes for 5 min each and a third wash for 1 min. (2) 100% ethanol, two washes, 10 min each; (3) 95% ethanol, 1 wash for 2 min; (4) 80% ethanol, 1 wash for 2 min; (5) 70% ethanol, 1 wash for 2 min; (6) Immersion in double distilled water (DDW).

To perform antigen retrieval, slides were treated with citrate buffer, pH 6 (HIER Citrate buffer pH = 6; Zytomed Systems; Germany) by microwave for 15 min (P100 for 3 min, P10 for 12 min). Slides were then cooled down and equilibrated to RT for 40 min and subsequently washed twice with wash buffer (Zytomed systems) for 4 min with gentle agitation.

Then the tissue on each slide was blocked using a hydrophobic barrier pen (Pink PAP pen, Zytomed system). For MCs staining, primary anti-tryptase antibody (Monoclonal Mouse Anti-Human Mast Cell Tryptase Clone AA1 Cat. No. M7052, Dako, USA) was diluted to the optimal concentration of 1:500 (determined via calibration experiments) in a blocking solution (Background blocker, BioSB, Germany), and applied to the tissue. The slides were then incubated in a humidity chamber overnight at 4 °C. Four control slides were incubated with mouse IgG1 isotype control (Cat. No. 14471482, eBioscience, ThermoFisher USA) at the same dilution as the anti-tryptase primary antibody (1:500).

The following day, slides were allowed to equilibrate to RT for 30 min. Then, they were washed twice with wash buffer for 4 min, with gentle agitation. Secondary antibody polymer conjugated to alkaline phosphatase (AP) (Zytochem Fast (AP) One-Step Polymer anti-Mouse/Rabbit/Rat, Cat. No. ZUC068-006 Zytomed) was added to the slides and incubated for 30 min at RT. Slides were then washed twice with wash buffer for 4 min with gentle agitation.

To visualize the enzymatic reaction ALK magenta chromogen (Polydetector Alk magenta, Goleta, CA, USA) was added to the slides and incubated for 20 min at RT. Slides were then submerged in DDW to stop the enzymatic reaction. Subsequently, slides were dipped quickly in hematoxylin (Gills Hematoxylin, Sigma Aldrich, Israel) and then dehydrated in the following solutions: (1) 95% ethanol for 2 min; (2) 100% ethanol twice for 1 min; (3) 50:50 ethanol:xylene for 1 min; (4) absolute xylene, tree times for 1 min each. Slides were then mounted using xylene based mounting medium (xylene based mounting medium; Bar Naor, Petah Tikva, Israel) and cover glass.

### Immunohistochemistry (IHC) staining for eosinophils

Eosinophils were stained using the same protocol described above for MCs with different primary and secondary antibodies. For eosinophils staining, anti-EPX primary antibody (mouse monoclonal anti-human/mouse EPX, IgG2a, Clone MM25-82.2, J. Lee labs, Mayo Clinic, Arizona, USA) was diluted to the optimal concentration of 1:200 (determined via calibration experiments) in a blocking solution (Background blocker, BioSB, Germany), was applied to the tissue, and then the slides were incubated in a humidity chamber overnight at 4 °C. Control slides were incubated with mouse IgG2a isotype control (Negative Control Mouse IgG2a Cat.No. X 0943, Dako, USA) at the same dilution as the anti-EPX primary antibody (1:200). The following day, slides were allowed to equilibrate to RT for 30 min. Then, they were washed twice with wash buffer for 4 min with gentle agitation. Goat anti–mouse AP conjugated secondary antibody (Goat anti–mouse IgG H&L Alkaline Phosphatase; ab97020, Abcam, Israel) was added to the slide at optimal concentration of 1:200 and incubated for 60 min at RT. Slides were then washed twice with wash buffer for 4 min with gentle agitation.

### Samples analyses

The integrity of the staining was examined and compared to isotype control samples by a pathologist. H&E samples were analyzed manually at ×400 magnification (high power field; hpf) using Nikon TL microscope (Core Research Facility, Faculty of Medicine, The Hebrew University of Jerusalem, Israel) whilst being blinded to the status of the patients. IHC staining of eosinophils and MCs was analyzed manually by an independent researcher blinded to the status of the patients at ×400 magnification using Olympus light microscope (School of Pharmacy, Faculty of Medicine, The Hebrew University of Jerusalem). Numerical values were assigned to each level of MCs and eosinophils count in the tissue as shown in Table 2. The total score for each sample was calculated as the average score from 5 fields with the maximal cell presence.

**Table 2:** Numerical values assigned to each level of tumor Eos/MCs count in each hpf

Level	Number of cells in hpf field
0	< 6
1	6–26
2	> 60

### RNA extraction

RNA extraction was performed using standard RNeasy kit for RNA purification (Qiagen, Hilden, Germany). Using a blade, tissue sections were scraped in tubes and immersed in 500 µl mineral oil for 10 min at 80 °C. Samples were then centrifuged (14,000 g, 1 min), washed with 1 ml ethanol and vortex for 15 s. Ethanol was then removed and samples were incubated in 180 μl protease K digestion buffer and 20 μl Proteinase K for 3-12 h at 55 °C. Then, 750 μl Trizol was added directly to the samples and incubated for 5 min and RT for lysis.

Then after, RNA was precipitated by adding 200 μl chloroform for 2–3 min at RT, and centrifuged at 12,000 g for 15 min, 4 °C. The upper aqueous phase was then collected to a new tube and an equal volume of 100% isopropanol was added. Samples were incubated for 10 min, centrifuged (12,000 g, 10 min, 4 °C) and the supernatant was discarded. The RNA pellet was then washed with 700 µl of 70% ethanol, centrifuged (12,000 g, 5 min, 4 °C), and the RNA pellets resuspended in 50 µl RNase-free water.

### RNA-sequencing of patient’s samples

Quality-trimming and trimming of poly-A sequences and adapters was done with Cutadapt [29] (version 2.10). Then, reads were filtered using the fastq_quality_filter program of the FASTX package, to keep only reads with a quality threshold of at least 20 at 90% of the read. Then these were mapped to the *Homo sapiens* transcriptome and genome (GRCh38 with annotations from Ensembl release 99) using TopHat [30] (version 2.1.1). Quantification of raw counts was done using htseq-count [31] (version 0.12.4). Genes were filtered, retaining only those genes for which a minimum of three of the samples had an expression value of 1 count per million. Subsequent differential expression analysis was done with the DESeq2 package [32]. Default parameters were used, except for not using independent Filtering algorithm. Significance threshold was taken as *p*adj < 0.1 (default).

RNA sequencing data were deposited in GEO database (GEO accession numbers: GSE291192).

### Gene set enrichment analysis (GSEA)

Whole differential expression data were subjected to gene set enrichment analysis using GSEA [33] to determine whether a priori defined set of genes showed statistically significant concordant differences between two biological states. We used the hallmark gene set collection, taken from the molecular signatures database (MSigDB [33]), as well as the REACTOME and the NAKAJIMA pathways database and a manually curated gene set collection related to immune cells and functions. Gene sets with FDR (corrected *p* value < 0.05) were considered significant.

### Statistical analysis

All statistical analyses were performed in GraphPad Prism software with data from three or more biologically independent replicates using two-tailed Student’s *t*-test or one-way ANOVA with Tukey’s multiple comparisons. Results are represented as mean ± standard deviation (SD). Statistical analyses were performed using GraphPad Prism 10.2.3.403 (GraphPad, San Diego, CA). The *p* values are *p < 0.05, **p < 0.01, ***p < 0.001, and ****p < 0.0001, NS-non-significant (*p* > 0.05).

## Supplementary Information

Below is the link to the electronic supplementary material.

Supplementary file1 (DOCX 9197 kb)

## Data Availability

The datasets used and/or analyzed during the current study are available from the corresponding author. RNA sequencing data were deposited in GEO database (GEO accession numbers: GSE291192).
