# Supplementary material for: Eosinophils but not mast cells exert anti-tumorigenic activity, without being predictive markers of the long-term response to Bacillus Calmette-Guérin (BCG) therapy in patients with bladder carcinoma
Source: Inflamm Res. 2025 Apr 24;74(1):68. doi: 10.1007/s00011-025-02028-1 (PMC12021714; doi:10.1007/s00011-025-02028-1)
Supplement: Supplementary file 1 — Supplementary file1 (DOCX 9197 kb) [file 11_2025_2028_MOESM1_ESM.docx]

# Eosinophils but not mast cells exert anti-tumorigenic activity, without being predictive markers of the long-term response to Bacillus Calmette-Guérin (BCG) therapy in patients with bladder carcinoma.

Ilan Zaffran^1^, Yara Zoabi^1^, Pratibha Gaur^1^, Fidan Rahimli Alekberli^1^, Ekaterini Tiligada^1,2^, Vladimir Yutkin^3#^, and Francesca Levi-Schaffer^1^**^#^**

**Supplemental figures:**

**Figure 1. (A)** RT112 cell viability assessed by MTT. RT112 cells co-cultured with pbEos at indicated ratios for 72h; n=3. **(B)** Catheter-delivered instillation imaging and bioluminescence analysis (IVIS) of mice following bladder cancer inoculation.

**Figure 2. (A-B)** Gene Set Enrichment Analysis (GSEA) of innate immune system (A) and extracellular matrix formation related-gene pathways (B; e.g extracellular matrix degradation, collagen formation and degradation) in responder vs. non-responder patients. All these pathways are up-regulated in responders, with FDR (corrected p-value) <0.05.

**Figures**

**Figure 1**

**A.**


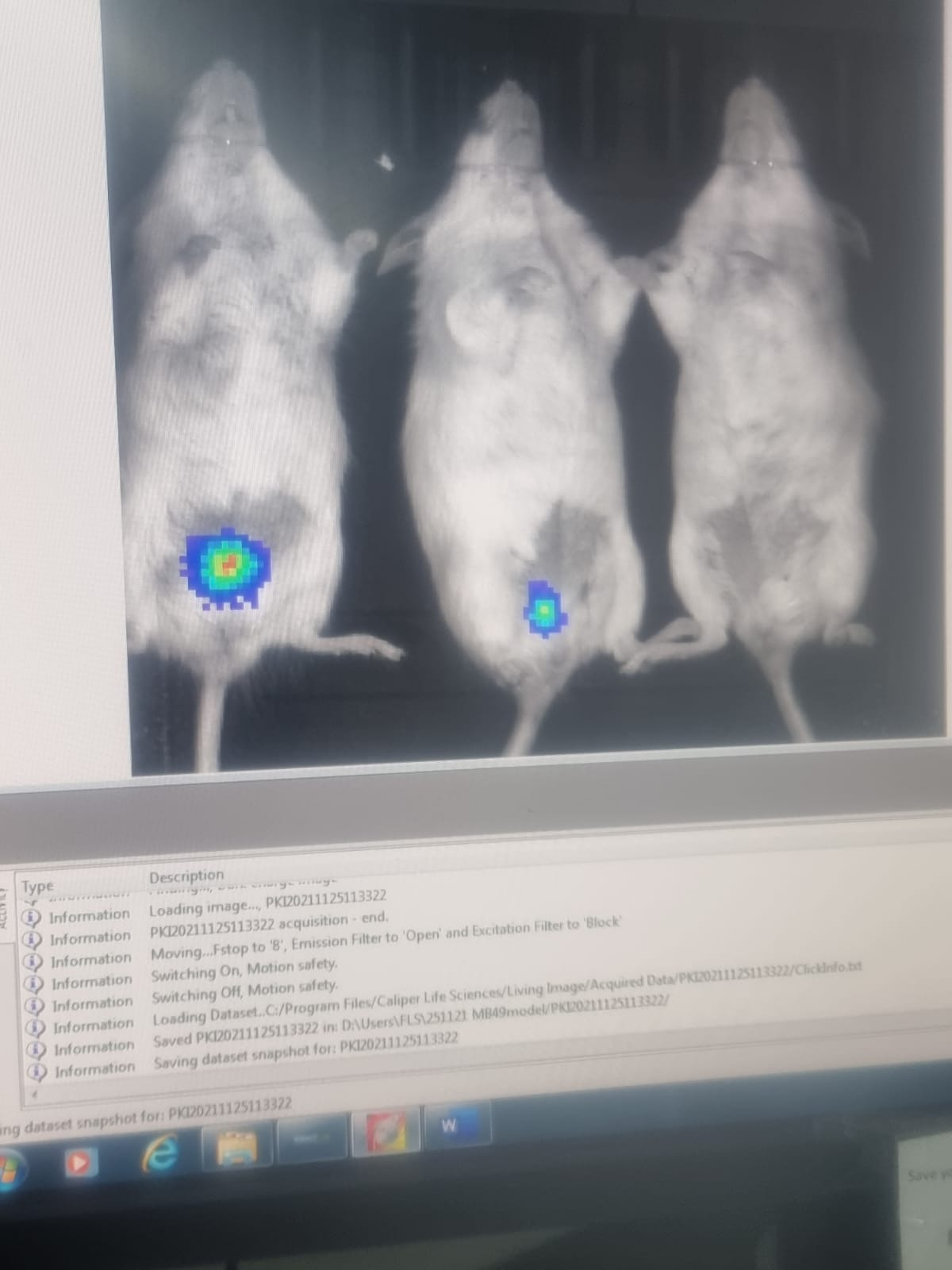


**B.**


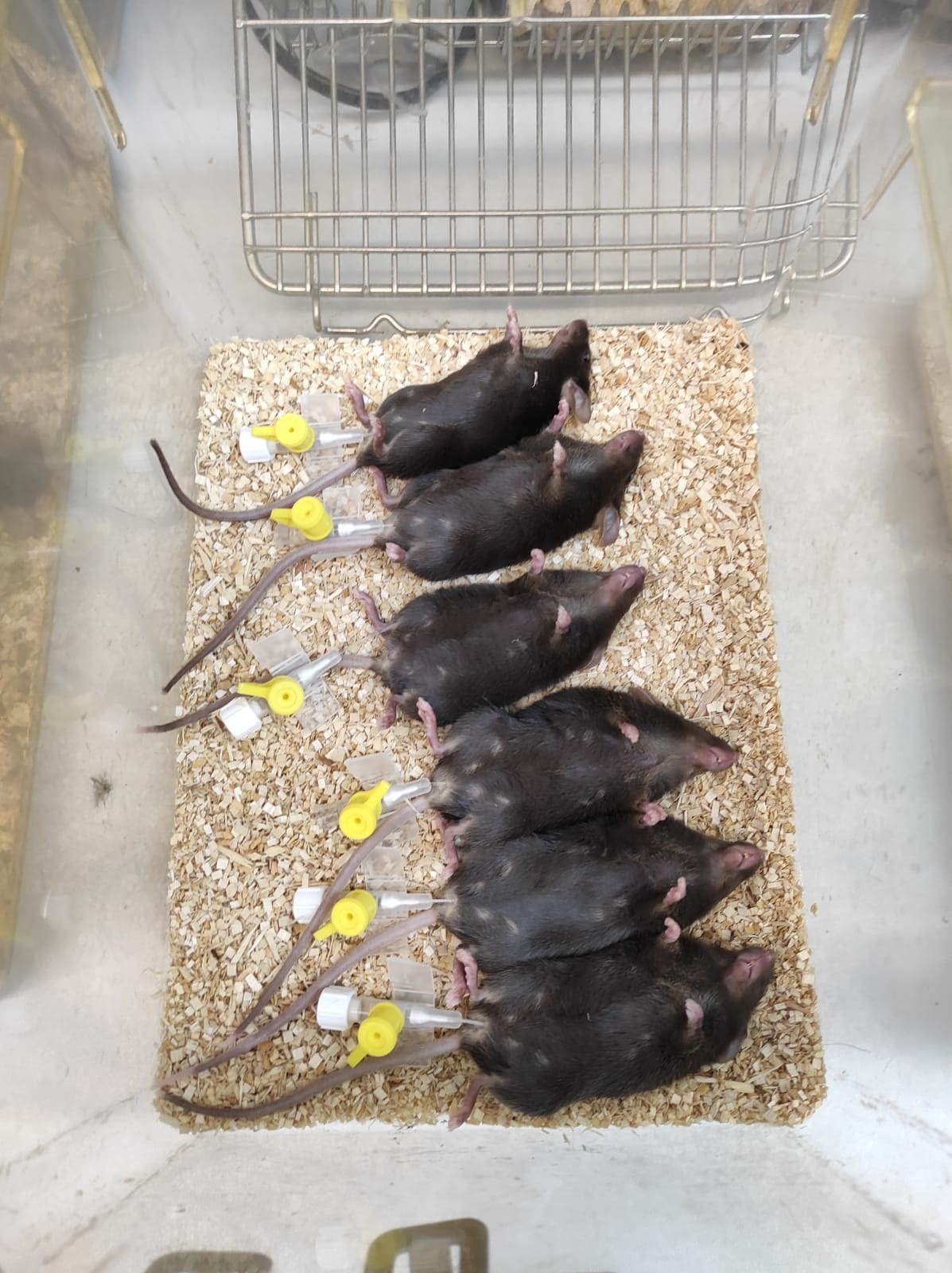


**Figure 2**

**
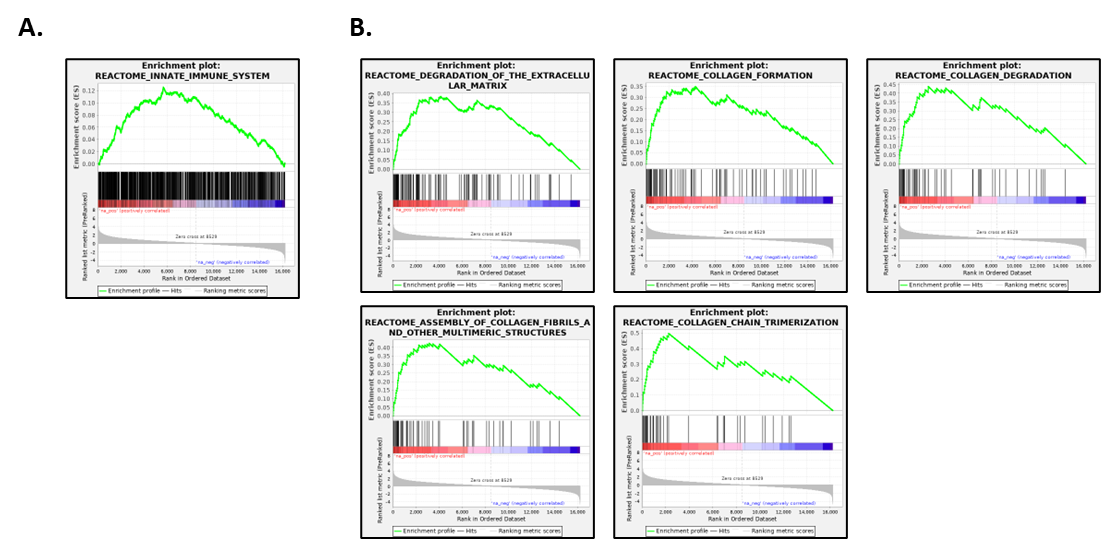
**
